# Neutrophil PAD4 Expression and Its Pivotal Role in Assessment of Alcohol-Related Liver Disease

**DOI:** 10.3390/ijms25147597

**Published:** 2024-07-11

**Authors:** Anna Rycyk-Bojarzynska, Beata Kasztelan-Szczerbinska, Halina Cichoz-Lach, Agata Surdacka, Jacek Rolinski

**Affiliations:** 1 Department of Gastroenterology with Endoscopy Unit, Medical University of Lublin, 20-090 Lublin, Poland; 2 Department of Clinical Immunology, Medical University of Lublin, 20-093 Lublin, Poland

**Keywords:** alcohol-related liver disease (ALD), alcohol-related liver cirrhosis (ALC), NETosis, neutrophil extracellular trap (NET), neutrophils, peptidyl arginine deiminase (PAD), peptidyl arginine deiminase 4 (PAD4)

## Abstract

Neutrophils release neutrophil extracellular traps (NETs) as a defense strategy in response to broad-spectrum infections and sterile triggers. NETs consist of a DNA scaffold decorated with antimicrobial peptides (AMPs) and enzymatically active proteases, including peptidyl arginine deiminase type 4 (PAD4). Susceptibility to infections and inflammatory dysregulation are hallmarks of alcohol-related liver disease (ALD). Sixty-two patients with ALD were prospectively recruited, and they were followed for 90 days. Twenty-four healthy volunteers served as the control group. PAD4 concentrations were quantified using immunoenzymatic ELISAs. Correlation coefficients between PAD4 blood concentrations and markers of systemic inflammation; liver dysfunction severity scores; and ALD complications were calculated. The receiver operating curves (ROCs) and their areas under the curve (AUCs) were checked in order to assess the accuracy of PAD4 expression in predicting the degree of liver failure and the development of ALD complications. Systemic concentrations of PAD4 were significantly increased in the patients with ALD in comparison with controls. PAD4 levels correlated with the standard markers of inflammation and revealed a good predictive AUC (0.76) for survival in the whole ALD group. PAD4 seems to be an inflammatory mediator and may be potentially applied as a predictor of patient survival in ALD.

## 1. Introduction

Almost 3.3 million people die every year as a consequence of harmful alcohol consumption [1]. Excessive alcohol consumption leads to the damage of multiple organs, including liver injury. Alcohol-related liver disease (ALD) leads to hepatocellular carcinoma (HCC) in about 30% of patients. Furthermore, it is very often found late because of already coexisting advanced liver disease, which makes it difficult to detect [2]. Overall, almost 25% of liver cirrhosis cases are caused by alcohol consumption [3]. ALD includes steatosis, fibrosis, cirrhosis, and hepatitis [4]. The liver is the crucial organ in the metabolism pathways of ethanol. Firstly, ethanol is metabolized into acetaldehyde and then to acetate in the oxidative pathway with alcohol dehydrogenase (ADH) and acetaldehyde dehydrogenase (ALDH), respectively. This process leads to increased transforming growth factor (TGF)-beta signaling, inflammation, fibrosis, and hypoxia of the hepatocytes. Subsequently, pro-inflammatory factors are activated, such as reactive oxygen species (ROS), cytokines, and neutrophils [3]. During ALD, neutrophils are recruited to the liver sinusoids and migrate to the liver parenchyma. These cells are the most abundant of all leukocytes and account for up to 75% of all circulating white blood cells (WBCs). During infection, neutrophils are the first-line key players to contribute to healing and recovery. They play a crucial role in adaptive immunity [5,6]. Brinkmann et al. showed that bacteria can be trapped and killed by neutrophils through neutrophil extracellular traps (NETs) as a host’s defense mechanism [7]. However, uncontrolled NET formation causes damage to healthy cells.

**Figure 1:**
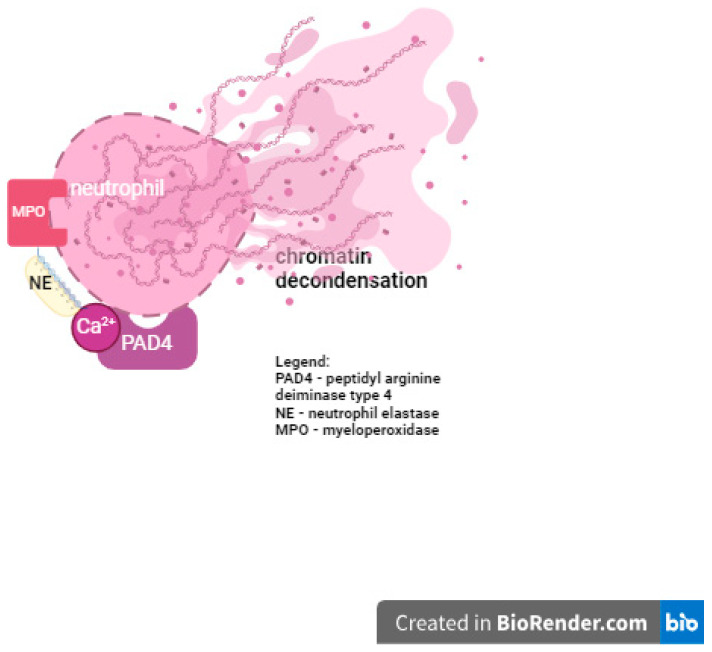
PAD4’s role in NETosis.

There are many different stimuli that can contribute to the process of NET formation, which is called NETosis. NETs are composed of chromatin and antibacterial proteins, including neutrophil elastase (NE), myeloperoxidase (MPO), matrix metalloproteinase 9 (MMP9), and cathepsin G (CG) [8]. The main process in NET formation is the decondensation of chromatin, which requires specific enzymes to be activated, including peptidyl arginine deiminase type 4 (PAD4) enzyme. The activation of PAD4 leads to the rupture of the nucleus and plasma membranes. Hence, it results in neutrophil death and is called “suicidal NETosis”. Another mechanism of NETosis is called “vital NETosis”, and its main feature is activation by Ca^2+^-activated PAD4 [9]. This mechanism results in reactive oxygen species (ROS)-independent NET formation [10,11]. NETosis is driven by PAD4 in an NADPH oxidase-dependent manner. As mentioned above, PAD4 appears to be crucial for chromatin decondensation. However, some other factors may be needed to contribute in a specific phase of decondensation, such as myeloperoxidase (MPO) and neutrophil elastase (NE). Figure 1 shows the schematic action of PAD4 during NETosis [9,12].

During a posttranslational modification, PAD4 converts arginine into citrulline, which is called citrullination. This process is catalyzed by enzymes from the peptidyl arginine deiminase (PAD) family. Notably, the activation of PAD4 leads to the formation of NETs. However, uncontrolled PAD4 activation is a driver of autoimmune diseases through autoimmune antibody formation. Therefore, the citrullination of histones by PAD4 suggests its role as a regulator of transcription [6,10,12]. PAD4 targets histones in the nucleus and cytoplasm of neutrophils, tumor cells, and macrophages. To the best of our knowledge, this is the only enzyme among the PAD family that is able to target histones through standardized nuclear localization sequence (NLS) [11]. Unfortunately, many pathways essential for NETosis are not completely understood yet. We hypothesized that PAD4 might be increased in the course of ALD and have an enormous influence on ALD assessment. Furthermore, PAD4 seems to be an inflammatory mediator as a consequence of systemic inflammation, as well as local inflammation in ALD patients. Interestingly, we believe that PAD4 may be potentially used as a predictor of patient survival in ALD.

## 2. Results

### 2.1. Study and Control Group

During the period of 90 days of observation, three patients died. As mentioned above, 86 consecutive patients were included in the study. Briefly, 62 patients with ALD, including 51 males with a mean age of 48.94 ± 10.29 and 11 females with a mean age of 47.78 ± 12.22, served as the studied group. The control group consisted of 24 age- and sex-matched healthy volunteers, including 15 males with a mean age of 46.21 ± 11.23 and 9 females with a mean age of 45.11 ± 10.23 who consumed no more than 10 g of ethanol daily. The analysis of parameters such as the alanine aminotransferase (ALT) level, aspartate aminotransferase (AST) level, ɣ-glutamyl-transpeptidase (GGT) level, international normalized ratio (INR), C-reactive protein (CRP) level, white blood cell (WBC) count, neutrophil (NEU) level, lymphocyte (LYM) level, NLR –NEU/LYM ratio, bilirubin level, albumin level, and creatinine level was performed among patients with ALD based on their gender, which is summarized in Table 1.

The results showed no correlation between the measured variables based on patient gender. In the control group, these variables were observed to be in normal ranges. The PAD4 concentrations were measured in the patients with ALD and in the control group patients. Table 2 shows the comparison of PAD4 concentrations between the ALD patients and the controls.

The PAD4 plasma concentrations were increased in the ALD patients, while in the controls, they were significantly lower (*p* = 0.0009).

### 2.2. Gender-Based Comparison

Further analysis of PAD4 concentrations in the ALD patients based on gender showed no significant differences between the ALD females and males. The data are shown in Table 3.

The next analysis was regarding patient gender depending on the studied group or the control group. Table 4 shows the comparison of PAD4 concentrations between the ALD females versus the control group females as well as the ALD males versus the control group males. (Relevant results are highlighted with green color).

Significant gender-related differences were observed among females. The ALD females presented with increased PAD4 concentrations compared to the control group females (*p* = 0.009), while these differences were not observed among the male population.

### 2.3. The Severity of Liver Failure

Our study revealed no correlation between PAD4 concentrations and the severity of liver failure classified by Child–Turcotte–Pugh score (class A, B, and C). The results are presented in Table 5.

Moreover, no significant correlation was observed between PAD4 concentrations and the MELD-Na score, which is shown in Table 6.

The severity of liver failure according to the mDF score also had no statistically significant correlation with PAD4 concentrations (as presented in Table 7).

**Figure 2:**
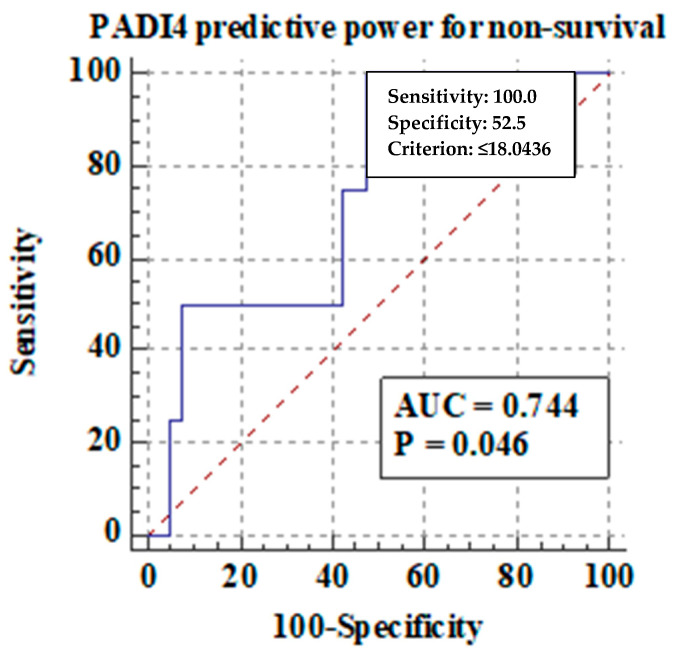
The ROC of PAD4 serum concentrations for predicting non-survival in the ALD patients.

Figure 2 shows the receiver operating characteristic (ROC) for PAD4 serum concentrations in the ALD patients for predicting non-survival.

PAD4 concentrations showed positive predictive area under curve (AUC) values for predicting non-survival in the studied group (AUC = 0.74, *p* = 0.04). No correlation was observed between PAD4 concentrations and symptoms of ALD decompensation, such as ascites, HE, EV, and kidney failure. Subsequently, the Spearman’s rank correlation test was used for the assessment of the association between indicators of inflammation (CRP, WBC, NEU, NLR) and PAD4 plasma concentrations in the ALD patients. The results are shown in Table 8.

**Figure 3:**
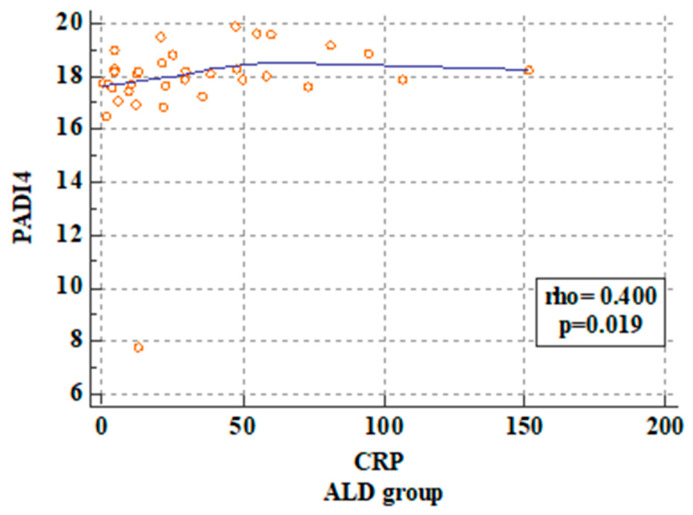
Correlation between PAD4 serum concentrations and CRP level in the ALD patients.

**Figure 4:**
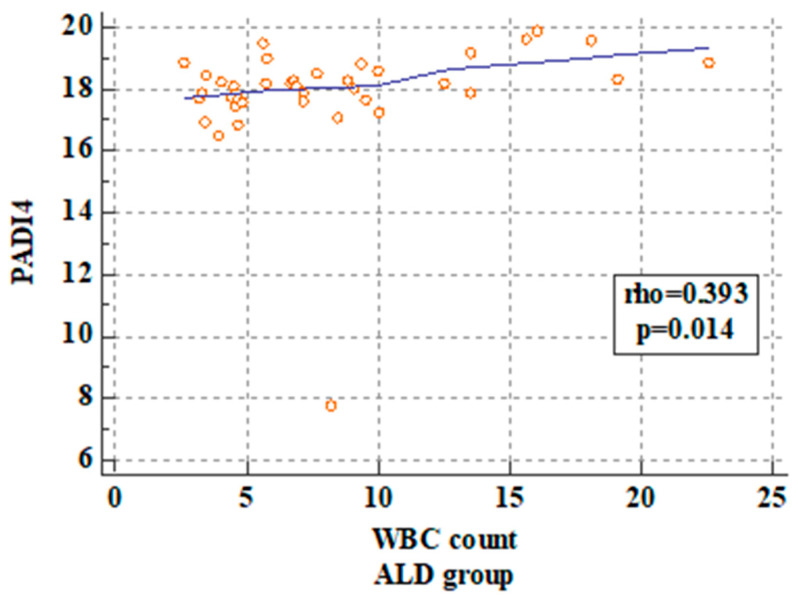
Correlation between PAD4 serum concentrations and WBC count in the ALD patients.

**Figure 5:**
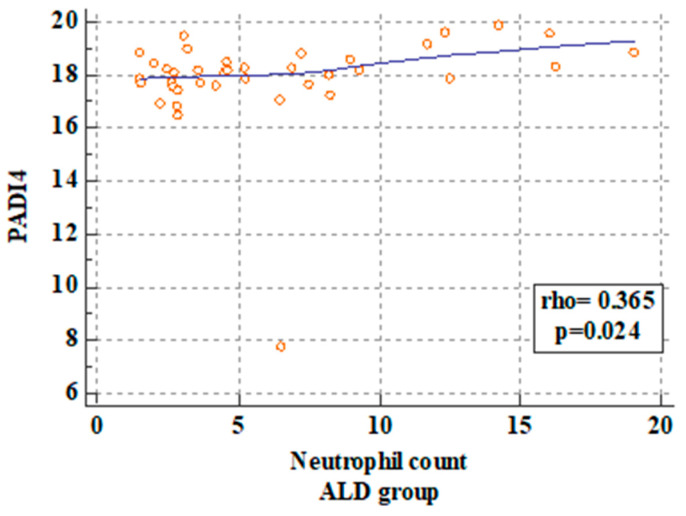
Correlation between PAD4 serum concentrations and neutrophil level in the ALD patients.

The statistical analysis revealed a significant correlation between PAD4 concentrations in the ALD patients and WBC, NEU, and CRP levels. The p value was 0.01, 0.02, and 0.01, respectively. These correlations are presented in Figure 3, Figure 4 and Figure 5.

Furthermore, the analysis of WBC count revealed that in the ALD patients with WBC count > 15 cells/ µL, increased PAD4 concentrations were observed compared to the ALD patients with WBC count ≤ 15 cells/µL (*p* = 0.0017). The results are presented in Table 9.

## 3. Discussion

### 3.1. PAD4 in Liver Disease

Collectively, our results confirm the fact that alcohol consumption contributes to revealing systemic inflammatory processes that may cause liver injury. Our data suggest that NET formation promotes inflammation in the injured liver, and PAD4 seems to be essential for NETosis. Although it is known that NET formation is related to PAD4, ROS, and histone citrullination, the whole mechanism remains unclear [13]. In the study, a population of ductular reaction-associated neutrophils (DRANs) was described. DRANs, which are new players in chronic liver injury, remain in direct contact with biliary epithelial cells in chronic liver disease. PAD4-deficient neutrophils inhibited neutrophil-induced biliary cell proliferation in mice [14].

As researchers have confirmed, the inhibition of NETosis leads to better survival among patients with different autoinflammatory diseases [15]. However, previous data are based on the mortality of mice, which was confirmed to be related to NET formation [13]. In our study, we revealed that significant gender-related differences in PAD4 were observed among females only. Probably, this is the consequence of female sex as a risk factor for a worse outcome of ALD, progression to cirrhosis, and alcoholic hepatitis [16]. To our knowledge, this is the first study showing gender-related differences in NET formation. We did not find any data regarding PAD4 levels in alcohol-related liver disease. Our study is the first one to check PAD4 levels in patients with ALD.

### 3.2. PAD4 in Sepsis

Previously, PAD4 was confirmed to be present in NETosis occurring in sepsis [17,18]. As Li et al. showed, the inhibition of PAD suppressed citrullinated histone H3 activity and improved the survival rates in septic mice [18]. On the other hand, according to Martinod et al., NETs are not responsible for bacterial infection-induced liver injury. They did not observe an increased mortality in sepsis in the PAD4 gene deletion mice [19]. The above differences in the results of the studies can be a consequence of the model procedure, control of mortality rates, and PAD4 inhibition to PAD4 gene deletion inequalities [20]. However, in the study in humans, PAD4 concentrations were associated with increased mortality when adjusted for APACHE II score and lactate (*p* < 0.001) [17]. In the study by Sun et al., PAD4 deficiency induced endoplasmic reticulum (ER) stress activation, while the inhibition of ER stress activation impaired intestinal apoptosis during sepsis. Their data show that NETs play a crucial role in severe sepsis. In PAD4-deficient mice, ER stress biomarkers were decreased in septic shock models [21]. The latest data demonstrated that in septic rats, the reduced expression of PAD4 in neutrophils might improve coagulation dysfunction in sepsis and inhibit the formation of NETs [22].

### 3.3. PAD4 in Rheumatoid Arthritis

Interestingly, in the PAD family, except for PAD4, PAD2 also seems to be an interesting enzyme, especially because of the fact that it is highly expressed in neutrophils and macrophages. The study by Tian et al. indicates that PADs other than PAD4 may be crucial in the pathogenesis of sepsis. Other studies show that PAD2 inhibition is beneficial in lupus, colitis, cancer, and hemorrhagic shock in animal models [23]. Both enzymes, PAD4 and PAD2, are present in synovial fluid in rheumatoid arthritis (RA) patients. Willis et al. revealed that Cl-amidine can transit membranes and inhibit PAD4 activity in the nucleus [24]. In the meta-analysis by Lee et al., PADI4_94 and PADI_104 polymorphisms were associated with susceptibility to RA in Asians and Caucasians [25,26]. Consistent with previous findings, PAD4-deficient neutrophils are unable to release NETs. In the study by van der Windt. et al., PAD4^−/−^ mice were naturally NET-deficient, and reduced levels of interleukin-6 (IL-6), TNF-α mRNA, and serum ALT were observed. The lack of PAD4 in mice led to a decrease in infiltrating macrophages [27]. In another study, mice with neutrophils lacking PAD4 displayed decreased apoptosis-associated speck-like (ASC) protein and NLR family pyrin domain containing 3 (NLRP3) inflammasome protein levels when compared with PAD4-positive neutrophils. Nowadays, NETosis is known to be regulated by PAD4. However, the role of NLRP3 remains unclear. In their study, PAD4 was essential for optimal NLRP3 and ASC protein levels post-transcriptionally. They showed PAD4-dependent formation of the NLRP3 inflammasome in neutrophils and the implication of NLRP3 in NETosis [28]. As PAD4 is thought to be a trigger of NETosis, several studies reveal its role in thrombosis, sepsis, cancer, and rheumatoid arthritis.

### 3.4. PAD4 in Cancer

Unfortunately, it remains unknown which factors are required for PAD4 activity. In vitro, calcium ionophores are used as key regulators of PAD4 activation. However, in vivo, other mechanisms seem to be crucial for PAD4 activation [10]. PAD4’s overexpression is observed in the tumor tissues and the blood of patients with cancer but exhibits low expression or even is not expressed in benign tumors as well as in normal tissues. PAD4 drives NETosis, promoting tumor growth and metastasis and resulting in poor prognosis. Overall, PAD4 can be helpful in the prevention of NETosis in cancer and be a useful potential treatment target [10,29,30]. Currently, there are some research studies that show the PAD4 potential drug effect. For example, simvastatin treatment reduces PAD4 expression and inhibits NETosis in mice with severe asthma [31]. In the study by Zhan et al., treatment with the PAD4 inhibitor (GSK484) results in significantly reduced pulmonary edema in mice by reducing levels of IL-6, TNF-α m RNA, CXC motif chemokine ligand 1 (CXCL1), and CXC motif chemokine ligand 2 (CXCL2) [32]. According to the present studies, in myocardial injury, the main source of toxic extracellular chromatin is PAD4-mediated NETosis [33]. The results obtained by Wong et al. showed that the inhibition of NETosis may improve wound healing in diabetes [34]. In another study, Perdomo et al. show that NETosis is present in mouse models with HIT (heparin-induced thrombocytopenia/thrombosis). Therefore, they suggest that NETosis is necessary to the thrombosis process. They focus on NET markers, including PAD4, and they indicate that PAD4-deficient mice injected with HIT antibodies and heparin do not develop any thrombus. Furthermore, clots are observed to be smaller in PAD4-deficient mouse models, suggesting that the size of the thrombus is also related to NETosis [35].

### 3.5. PAD4 in Colitis

As NETs were confirmed to be present in COVID infections, PAD4 inhibition may have a therapeutic effect on thrombotic and inflammatory mechanisms [36]. Interestingly, PAD4 inhibition is suggested to be promiscuous in colitis as well. Zhang et al. described that in colitis mice, there is an enhanced expression of citrullinated histone H3 (CitH3) and PAD4, which leads to the neutrophils’ NET production. On the other hand, the inhibition of NET formation through Cl-amidine improved the clinical colitis index in mice and reduced the gene expression of PAD4 in the colon, causing the inhibition of NET formation [37].

### 3.6. Proinflammatory Cytokines in NETosis

Calcium signaling, as we showed in Figure 1, seems to be essential for NET formation and cytokine secretion [38,39]. NETosis is a cytokine-dependent process in which IL-1β remains the main leader [40]. Furthermore, blocking the IL-1β receptor reduces NET formation. According to the study, IL-1β overproduction activates NLRP3 inflammasomes, leading to NET formation [41]. On the other hand, IL-8 is thought to be the most effective cytokine in NET formation [42]. Barbu et al. confirmed that NET formation is increased when IL-6 and IL-1ra levels are increased, too [43]. The newest cytokine that is elevated in rheumatoid arthritis is IL-40. IL-40’s release is raised by NET formation [44]. Interestingly, infliximab, which is a cytokine-neutralizing antibody, has been confirmed to reduce NET formation in patients with autoimmune disorders [45]. CRP level in NETosis was a subject of our study, as presented in Table 8.

### 3.7. Limitations of the Study

Unfortunately, our study has some limitations. Firstly, our study was an observational study. Next, 77% of the study population were males, which limited the study’s generalizability to both sexes. However, our study included all ALD patients who were admitted to the hospital. Overall, males more commonly have ALD compared to females.

## 4. Materials and Methods

Our study assessed 86 consecutive patients, including 62 patients with ALD who were prospectively recruited to the study over 2 years, and the controls. Fifty-one males and eleven females served as the studied group, with a mean age of 48.94 ± 10.29. The control group consisted of 24 age- and sex-matched healthy volunteers, including 15 males and 9 females who consumed no more than 10 g of ethanol daily, as suggested by the WHO [46]. Table 10 shows the characteristics of the study group and the control group.

We used the same patient recruitment protocol as presented in our previous studies [47,48,49,50].

### 4.1. Study Population

The diagnosis of ALD is based on “EASL Clinical Practice Guidelines: Management of alcohol-related liver disease” upon the documentation of regular alcohol consumption confirmed by the patients or their family members [1]. The AUDIT (Alcohol Use Disorders Inventory Test), which remains the ‘gold standard’ screening tool to identify alcohol use disorder (AUD), was abridged into the AUDIT-C (Alcohol Use Disorders Inventory Test-Consumption) [51,52,53,54]. In our study, the AUDIT-C was used in every patient, and 100% of patients from the studied group received at least 3 points, which was a positive test result. The AUDIT-C results were negative for all of the controls. The diagnosis of alcohol-related liver disease was based on symptoms, physical examination, laboratory abnormalities, and imaging tests in accordance with the European Association for the Study of the Liver (EASL) Clinical Practice Guidelines [1].

The studied patients were divided into subgroups based on the following:

(1) Gender;
(2) The severity of liver failure classified by the following:
  - Child–Turcotte–Pugh (CTP) score,
  - Model for End-stage Liver Disease-Sodium (MELD-Na) score,
  - Modified Maddrey Discriminant Function (mDF) score;
(3) ALD decompensation symptoms, such as ascites, HE, EV, and kidney dysfunction.

Certain diseases that can lead to liver injury must have been excluded, such as celiac disease, Wilson’s disease, alpha-1-antitrypsin deficiency, viral hepatitis, and autoimmune liver disease. The patients included in the study did not have any severe comorbidities (malignancy, respiratory failure, or cardiovascular diseases) and, during the previous 6 months, could not have had any blood transfusion, immunotherapy, or steroid treatment. The severity of liver failure was established using CTP, MELD-Na, and mDF scores using internet-available calculators. Kidney dysfunction was measured with the level of serum creatinine more than the upper limit of normal ranges (1.3 mg/dL).

### 4.2. ALD Decompensation

The patients with suspicion of alcohol-related liver cirrhosis underwent upper gastrointestinal tract endoscopy. Among the individuals with clinically significant portal hypertension (CSPH), which is recognized based on a hepatic vein pressure gradient (HVPG) of 10 to 12 mm Hg, esophageal varices (EV) occur [55]. The presence of portal hypertension was detected in Doppler-mode abdominal ultrasonography. It was observed in 58% of ALD patients. In our study, 45% of the studied patients were detected with EV. Performing a liver biopsy was not necessary to establish the diagnosis. Imaging techniques included abdominal ultrasonography or computed tomography (CT) scans [1]. Every patient from the studied group who presented the symptoms of hepatic encephalopathy (HE) was evaluated using the Clinical Hepatic Encephalopathy Staging Scale (CHESS) [56,57,58]. Eighteen studied patients were diagnosed with HE. The patients with ascites underwent diagnostic paracentesis to exclude spontaneous bacterial peritonitis (SBP). Large-volume paracentesis was necessary in 87% of ALD patients. According to the guidelines, after 5 L paracentesis, the patients were administrated with an albumin infusion [1]. Only 4 patients were diagnosed with SBP and received antibiotic treatment. The individuals were followed for 90 days. All studied patients were discharged from the hospital when liver function began to improve. Subsequent follow-up visits were every 2 weeks (during 90 days) in the outpatient clinic or in the hospital department if necessary.

### 4.3. Procedures

Venous blood samples were obtained from the individuals after overnight fasting upon hospital admission. All the tests were performed in the laboratory of the Department of Clinical Immunology at the Medical University of Lublin. The study was performed according to the manufacturer’s recommendations. Fifteen milliliters of peripheral blood was obtained from the ALD patients and the control group with the sterile S-Monovette (SARSTEDT AG & Co., D-51588 Numbrecht, Germany); subsequently, they were incubated for 20 min in a dark, dry room at room temperature. Plasma levels of PAD4 concentrations were quantified by an enzyme-linked immunosorbent assay (ELISA) with a PADI4/PAD4 ELISA kit (LS Bio; Biotech Company, Lynnwood, WA 98036, USA).

### 4.4. Statistical Analysis

Statistical analysis of the results was performed with the use of the Statistica 10 software package (StatSoft, Kraków, Poland). The deviation from normality was evaluated by the Kolmogorov–Smirnov test. The Mann–Whitney U test was used for between-group comparisons, while Spearman rank correlation was used to verify the correlations between inflammation markers and PAD4 concentrations. The categorical variables were compared using either the Fisher’s exact test or the χ^2^. The differences in PAD4 concentrations between CTP classes were analyzed using the Kruskal–Wallis test. The receiver operating characteristic (ROC) curves and the area under the curve (AUC) values were checked in order to assess the sensitivity and specificity of the measured variables in predicting the degree of liver failure. The method of DeLong was used for the calculation of the standard error of the AUC. The Youden index was estimated for marking optimal points. A value of p less than 0.05 was considered to be statistically significant.

### 4.5. Ethical Requirements

According to the Helsinki Declaration, all patients signed an informed written consent. The local ethics committee of the Medical University of Lublin approved the study (No. KE-0254/94/2019).

## 5. Conclusions

In conclusion, all the abovementioned studies were performed on mice and rats. Our study seems to be unique because we used human peripheral blood and measured PAD4 concentrations in ALD patients. However, there are not many data regarding chronic liver disease. To the best of our knowledge, PAD4 was not previously researched according to alcohol-related liver disease. Our data are valuable because the only therapeutic chance for patients with ALD that remains is a liver transplant. Over the last few years, the rates of ALD have increased rapidly. Inflammatory response inhibition seems to be the best possible treatment option for the near future. However, the mechanisms of immunological pathways remain unclear [59]. In this study, further PAD4 analysis has been achieved to indicate its value as a biomarker in ALD assessment. Furthermore, PAD4 seems to be useful as an inflammatory mediator. Consistently, we reported that PAD4 might be used as a predictor of patient survival in ALD. We consider that further studies on the PAD enzyme family could broaden the range of non-invasive inflammatory markers in the course of other diseases, including alcohol-related liver disease.

## Figures and Tables

**Table 1:** Demographic and laboratory data in patients with ALD.

Variable	Males ALD *n* = 51		Females ALD *n* = 11		*p*
	Median	5–95 Percentile	Median	5–95 Percentile	
Age (years) ^a^	47.00	33.00–64.00	56.00	26.00–61.00	0.28
ALT (U/L) ^a^	42.00	17.50–228.25	38.00	22.00–480.00	0.93
AST (U/L) ^a^	101.00	34.40–360.40	112.00	43.00–550.00	0.74
ALP (U/L) ^a^	153.00	56.50–405.15	121.00	73.00–411.00	0.76
GGT (U/L) ^a^	378.00	43.20–2558.20	444.00	155.00–2193.00	0.16
Bilirubin (mg/dL) ^a^	2.90	0.60–16.57	1.70	0.60–34.10	0.82
Albumin (g/dL) ^a^	3.08	2.00–4.13	2.94	2.43–4.62	0.73
INR ^a^	1.29	0.93–2.23	1.33	0.60–2.54	0.87
Creatinine (mg/dL) ^a^	0.80	0.42–1.57	0.60	0.40–2.90	0.14
CRP (mg/L) ^a^	20.23	1.69–144.82	23.90	0.53–109.70	0.74
WBCs (×10^9^/L) ^a^	7.00	3.24–15.94	5.79	4.07–13.55	0.94
NEUs (×10^9^/L) ^a^	4.52	1.51–13.85	3.57	2.74–12.52	0.86
LYMs (×10^9^/L) ^a^	1.06	0.44–2.37	0.92	0.46–1.55	0.39
NLR ^a^	3.98	1.47–14.81	4.03	2.06–27.22	0.68
CTP (pkt.) ^a^	8.00	5.00–13.00	8.00	5.00–13.00	0.78
MELD-Na (pkt.) ^a^	15.00	6.25–25.00	11.00	7.00–39.00	0.89
mDF (pkt.) ^a^	24.40	2.21–75.39	15.40	1.06–102.44	0.88
Ascites, n (%) ^b^	26 (50.98)		5 (45.45)		0.65
Encephalopathy, n (%) ^b^	16 (31.37)		2 (18.18)		0.67
Esophageal varices, n (%) ^b^	24 (47.05)		4 (36.36)		1.00
Non-survival, n (%) ^b^	2 (3.92)		1 (9.09)		0.33

^a^ Mann–Whitney test; ^b^ Fisher test; ALD—alcohol-related liver disease, ALT—alanine aminotransferase, AST—aspartate aminotransferase, GGT—ɣ-glutamyl-transpeptidase, INR—international normalized ratio, CRP–C-reactive protein, WBCs—white blood cells, NEUs—neutrophils, LYMs—lymphocytes, NLR—NEU/LYM ratio, CTP—Child–Turcotte–Pugh score, MELD-Na—Model for End-Stage Liver Disease-Sodium score, mDF—modified Maddrey Discriminant Function.

**Table 2:** Comparison of PAD4 serum concentrations between study group and control group.

Variable (ng/mL)	ALD Patients *n* = 62		Controls *n* = 24		*p* ^a^
	Median	5–95 Percentile	Median	5–95 Percentile	
PAD4	18.00	16.53–19.53	17.00	11.50–18.51	0.0009

^a^ Mann–Whitney test; ALD—alcohol-related liver disease; PAD4—peptidyl arginine deiminase type IV.

**Table 3:** Comparison of PAD4 serum concentrations between the ALD patients based on patient gender.

Variable (ng/mL)	ALD Males *n* = 51		ALD Females *n* = 11		*p* ^a^
	Median	5–95 Percentile	Median	5–95 Percentile	
PAD4	18.06	16.24–19.59	17.92	17.28–18.24	0.74

^a^ Mann–Whitney test; ALD—alcohol-related liver disease, PAD4—peptidyl arginine deiminase type IV.

**Table 4:** Comparison of PAD4 serum concentrations between the study group and the control group based on patient gender.

Variable (ng/mL)	ALD Males *n* = 51		Males Control Group *n* = 15		*p*_1_ ^a^	ALD Females *n* = 11		Females Control Group *n* = 9		*p*_2_ ^a^
	Median	5–95 Percentile	Median	5–95 Percentile		Median	5–95 Percentile	Median	5–95 Percentile	
PAD4	18.06	16.24–19.59	17.19	12.04–19.09	0.028	17.92	17.28–18.24	16.83	0.00–18.27	0.009

^a^ Mann–Whitney test; ALD—alcohol-related liver disease, PAD4—peptidyl arginine deiminase type IV.

**Table 5:** Comparison of PAD4 serum concentrations in the ALD patients based on CTP class (A, B, and C).

Variable (ng/mL)	ALD												*p* ^a^
	CTP Class A ^a^ *n* = 17				CTP Class B ^a^ *n* = 25				CTP Class C ^a^ *n* = 20				
	Median	Minimum	25–75 Percentile	Maximum	Median	Minimum	25–75 Percentile	Maximum	Median	Minimum	25–75 Percentile	Maximum	
PAD4	17.93	16.49	17.56–18.44	19.88	18.11	7.73	17.64–18.56	19.61	17.95	16.83	17.29–18.46	19.55	0.95

^a^ Kruskal–Wallis test; ALD—alcohol-related liver disease, CTP—Child–Turcotte–Pugh score, PAD4—peptidyl arginine deiminase type IV.

**Table 6:** Comparison of PAD4 serum concentrations in the ALD patients based on MELD-Na score (>20 and ≤20 points).

Variable (ng/mL)	MELD-Na > 20 *n* = 13		MELD-Na ≤ 20 *n* = 49		*p* ^a^
	Median	5–95 Percentile	Median	5–95 Percentile	
PAD4	18.08	17.60–19.22	17.89	17.58–18.19	0.24

^a^ Mann–Whitney test; ALD—alcohol-related liver disease, MELD-Na—Model for End-Stage Liver Disease—Sodium score, PAD4—peptidyl arginine deiminase type IV.

**Table 7:** Comparison of PAD4 serum concentrations in the ALD patients based on mDF score (>32 and ≤32 points).

Variable (ng/mL)	mDF > 32 *n* = 26		mDF ≤ 32 *n* = 36		*p* ^a^
	Median	5–95 Percentile	Median	5–95 Percentile	
PAD4	18.08	17.53–18.44	17.84	17.58–18.19	0.40

^a^ Mann–Whitney test; ALD—alcohol-related liver disease, mDF—modified Maddrey Discriminant Function, PAD4—peptidyl arginine deiminase type IV.

**Table 8:** Correlations of PAD4 serum concentrations with inflammatory markers (CRP, WBC, NEU, NLR) in ALD patients.

Inflammatory Markers	PAD4	
	rho	*p* ^a^
CRP (mg/L)	0.40	0.01
WBCs (×10^9^/L)	0.39	0.01
NEUs (×10^9^/L)	0.36	0.02
NLR	0.19	0.26

^a^ Spearman’s rank correlation coefficient; PAD4—peptidyl arginine deiminase type IV, CRP—C-reactive protein, WBCs—white blood cells, NEUs—neutrophils, NLR—NEU/LYM ratio.

**Table 9:** Comparison of PAD4 serum concentrations in the ALD patients based on WBC count (>15 and ≤15 cells/µL).

Variable (ng/mL)	WBCs > 15 (cells/µL) *n* = 7		WBCs ≤ 15 (cells/µL) *n* = 55		*p * ^a^
	Median	5–95 Percentile	Median	5–95 Percentile	
PAD4	19.58	18.20–19.82	17.88	16.40–18.86	0.0017

^a^ Mann–Whitney test; WBCs—white blood cells, PAD4—peptidyl arginine deiminase type IV.

**Table 10:** Characteristics of the study group and the control group.

Group	Patients (*n*)	Description
Study group	62	Patients with alcohol-related liver disease (ALD)
Control group	24	Healthy volunteers with alcohol consumption no more than 10 g ethanol per day

## Data Availability

The data presented in this study are available on request from the corresponding author.
